# Cytotoxic Effects of Dillapiole on Embryonic Development of Mouse Blastocysts *in Vitro* and *in Vivo*

**DOI:** 10.3390/ijms150610751

**Published:** 2014-06-13

**Authors:** Wen-Hsiung Chan

**Affiliations:** 1 Department of Bioscience Technology and Center for Nanotechnology, Chung Yuan Christian University, Chung Li 32023, Taiwan; E-Mail: whchan@cycu.edu.tw; Tel.: +886-3-265-3515; Fax: +886-3-265-3599.; 2 Center for Biomedical Technology, Chung Yuan Christian University, Chung Li 32023, Taiwan

**Keywords:** dillapiole, blastocyst, apoptosis, embryonic development

## Abstract

We examined the cytotoxic effects of dillapiole, a phenylpropanoid with antileishmanial, anti-inflammatory, antifungal, and acaricidal activities, on the blastocyst stage of mouse embryos, subsequent embryonic attachment and outgrowth *in vitro*, and *in vivo* implantation via embryo transfer. Blastocysts treated with 2.5–10 μM dillapiole exhibited a significant increase in apoptosis and corresponding decrease in total cell number. Notably, the implantation success rates of blastocysts pretreated with dillapiole were lower than those of their control counterparts. Moreover, *in vitro* treatment with 2.5–10 μM dillapiole was associated with increased resorption of post-implantation embryos and decreased fetal weight. Our results collectively indicate that dillapiole induces apoptosis and retards early post-implantation development, both *in vitro* and *in vivo*. However, the extent to which this organic compound exerts teratogenic effects on early human development is not known at present. Further studies are required to establish effective protection strategies against the cytotoxic effects of dillapiole.

## 1. Introduction

*Peperomia pellucida* is an annual herb belonging to the Piperaceae family, mainly located in Africa, Central and South America, Australia, and Southeast Asia. Traditionally, *P. pellucid* has been used as a food and medicinal herb [1]. *P. pellucida* is utilized as folk medicine for the treatment of gastric ulcer, along with several diseases, such as gout arthritis, wounds, high blood cholesterol, and skin-related problems (*i.e.*, acne) [2,3,4]. To date, plant-derived natural products have been mainly identified as playing critical roles for application in cancer chemotherapy strategies [5]. *Piper aduncum* is one particularly specie from the *Peperomia pellucid.* Dillapiole, a phenylpropanoid, is the main component of *Piper aduncum* [6,7], with reported antileishmanial [8], anti-inflammatory [9], antifungal [6], and acaricidal [10] bioactivities. However, the cytotoxic effects of this organic compound are not well documented at present. A recent study demonstrated that dillapiole exerts broad cytotoxic effects, with significant potential for development as an anticancer drug against a variety of tumor cell types. Further investigation disclosed that the compound acts as a pro-oxidant to induce intracellular reactive oxygen species in MDA-MB-231 cells [11]. Moreover, dillapiole inhibits cell proliferation through cell cycle arrest at the G_0_/G_1_ phase and association with disruption of actin filaments. These findings highlight the potential of dillapiole as a promising anticancer agent. Importantly, previous studies by our group led to the identification of natural chemical compounds that induce apoptosis and have hazardous effects on mouse embryonic development [12,13,14]. However, virtually no researchers have explored the potential cytotoxicity of dillapiole against embryos until now. In this study, we investigated the effects of dillapiole on pre-implantation and post-implantation stage embryos, with the aim of ascertaining its potential to impair embryonic development. Our results provide important information on the impact of dietary dillapiole that can be extrapolated to humans, in particular pregnant women.

During normal embryogenesis, apoptosis (a unique morphological pattern of cell death) functions to clear abnormal or redundant cells in preimplantation embryos [15,16]. Apoptotic processes do not occur prior to the blastocyst stage during normal mouse embryonic development [17], and induction of apoptosis during the early stages of embryogenesis (*i.e.*, following exposure to a teratogen) causes embryonic developmental injury [12,13,18,19,20]. Apoptosis plays an important role in development and disease [21]. Several reports have confirmed the significance of apoptosis in normal embryonic development [22,23,24]. However, excessive apoptosis triggered in early embryos by mechanistically diverse teratogens can lead to developmental injury [13,18,25,26,27]. Dillapiole has been shown to induce apoptosis in MDA-MB-231 cells by increasing oxidative stress via the mitochondrial pathway [11].

In the present study, we investigated whether the apoptotic inducer, dillapiole, has cytotoxic effects on embryonic development, using mouse blastocysts as the assay model. The effects of dillapiole on subsequent developmental injury of blastocysts *in vitro* and embryo transfer *in vivo* were additionally examined.

## 2. Results

### 2.1. Effects of Dillapiole on Mouse Blastocysts

**Figure 1:**
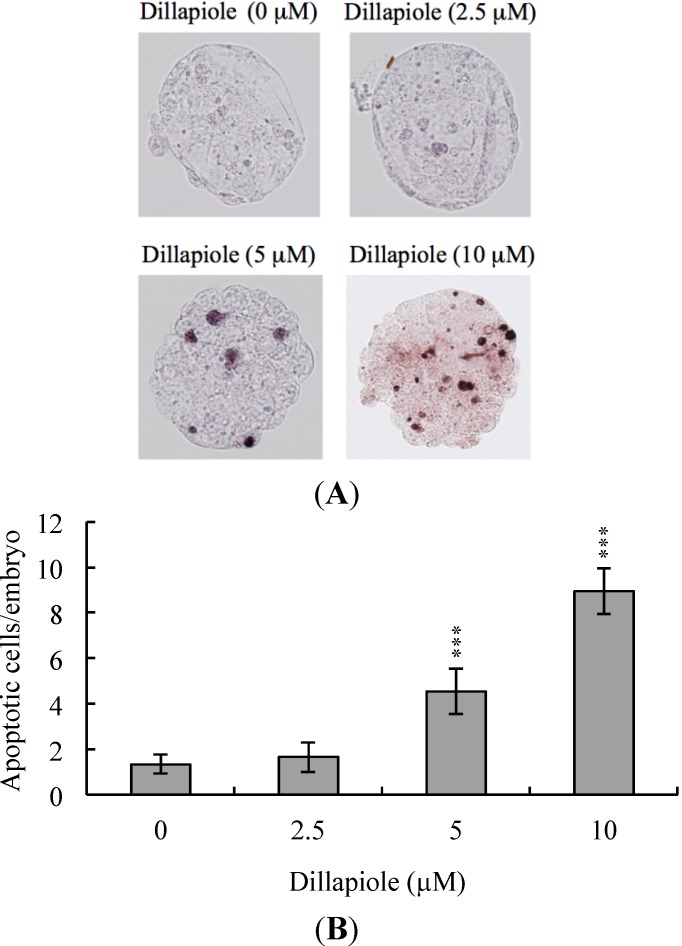
Dillapiole induces apoptosis in mouse blastocysts. (**A**) Mouse blastocysts were treated with dillapiole (2.5, 5, or 10 μM) for 24 h or left untreated, and apoptosis examined via TUNEL staining. Cells were visualized using light microscopy. TUNEL-positive cells are depicted in black; (**B**) The mean number of apoptotic (TUNEL-positive) cells per blastocyst was calculated. Data are based on at least 180 blastocyst samples from each group and presented as means ± SEM of six determinations. *** *p* < 0.001 *versus* the control group.

To determine whether dillapiole induces embryonic cytotoxicity, we treated mouse blastocysts with 2.5, 5, or 10 μM dillapiole at 37 °C for 24 h and examined DNA fragmentation, a characteristic of cell apoptosis, using the TUNEL assay. Apoptosis was evident in blastocysts treated with 5–10 μM dillapiole (Figure 1A). Quantitative analysis further revealed a nine-fold increase in apoptosis in 10 μM dillapiole-treated blastocysts, compared with untreated control cells (Figure 1B). Our results clearly signify that dillapiole triggers apoptosis in mouse blastocysts.

### 2.2. Effects of Dillapiole on Cell Proliferation

**Figure 2:**
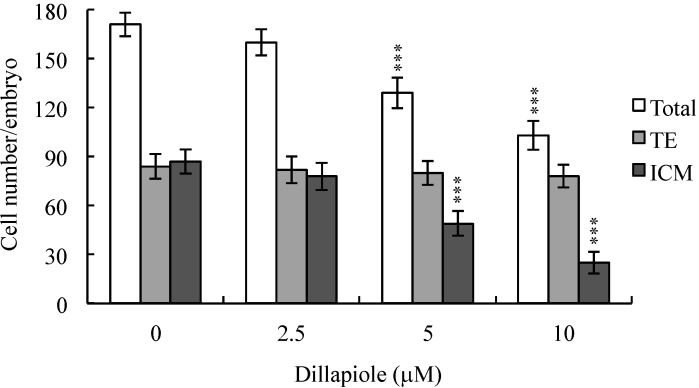
Effects of dillapiole on blastocyst viability. Mouse blastocysts were treated with dillapiole (2.5, 5, or 10 μM) or left untreated for 24 h, and differentially stained after treatment. Inner cell mass (ICM) cells are stained blue, and trophectoderm cells (TE) stained red. The total number of cells per blastocyst and cell numbers in the ICM and TE were counted. Data are based on at least 180 blastocyst samples from each group. *** *p* < 0.001 *versus* the control group.

To further determine the effects of dillapiole on embryo cell proliferation and establish whether inner cell mass (ICM) or trophectoderm (TE) cells or both blastocyst populations are most significantly affected, differential staining, followed by cell counting, was used to assess proliferation in blastocysts either treated with 2.5, 5, or 10 μM dillapiole or left untreated at 37 °C for 24 h. We observed significantly fewer ICM cells in blastocysts treated with 5–10 μM dillapiole, compared to control cells (Figure 2), implying marked inhibition of cell proliferation (Figure 2). The cytotoxic effects of dillapiole, in turn, impaired the developmental potential of blastocysts. Notably, dillapiole did not inhibit proliferation of TE cells of mouse blastocysts (Figure 2).

### 2.3. Effects of Dillapiole on Mouse Embryonic Developmental Potential in Vitro

**Figure 3:**
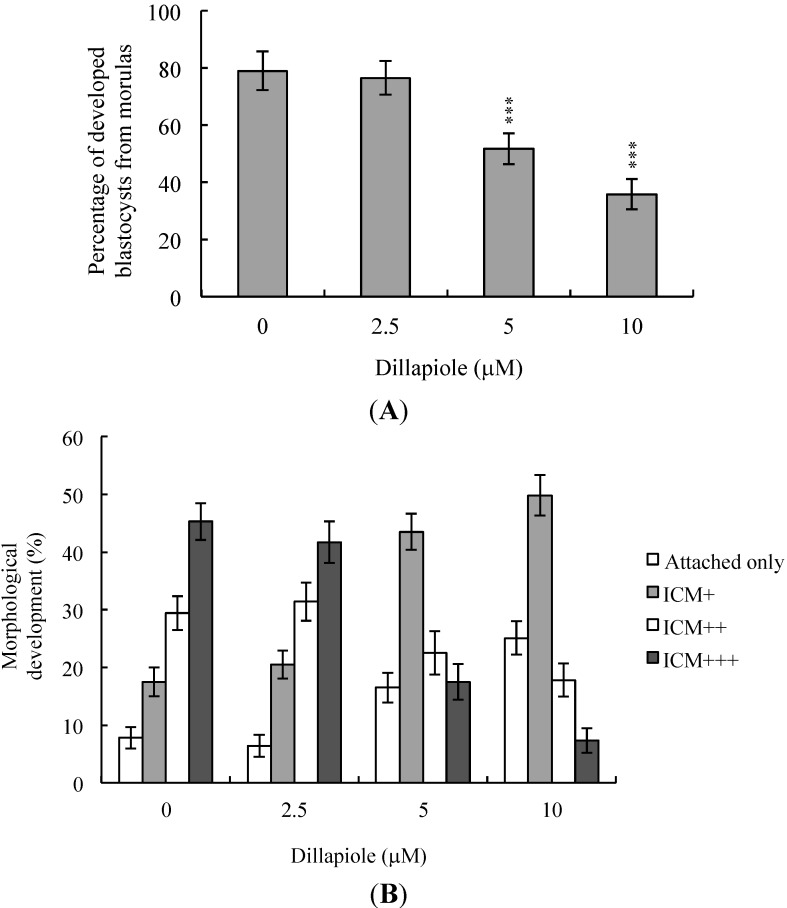
*In vitro* development of mouse embryos exposed to dillapiole at the blastocyst stage. (**A**) Mouse morulas were treated with dillapiole (2.5, 5, or 10 μM) or left untreated for 24 h and cultured for an additional 24 h at 37 °C. Blastocysts were counted and percentages calculated; (**B**) Mouse blastocysts were treated with dillapiole (2.5, 5,or 10 μM) or left untreated for 24 h, and cultured for seven days post-treatment. Blastocysts were classified as attached only, ICM+, ICM++, and ICM+++, based on morphological assessment, as described in Materials and Methods. Data are based on at least 200 blastocystsamples from each group and presented as means ± SEM of ten determinations. *** *p* < 0.001 *versus* the control group.

Further experiments to establish the effects of dillapiole on embryonic development potential from the morula to blastocyst stages revealed a lower proportion of dillapiole-treated morulas developing to blastocysts, compared to the untreated control group (Figure 3A). To determine the effects of dillapiole on post-implantation eventsn *in vitro*, blastocysts were treated with 2.5, 5, or 10 μM dillapiole or left untreated, and subsequent development analyzed for eight days in culture. The rate of embryo attachment to fibronectin-coated culture dishes and lack of further development (attachment only group) was markedly higher in dillapiole-treated blastocysts (Figure 3B). Additionally, pretreated blastocysts displayed a lower incidence of post-implantation developmental milestones (Figure 3B). Our results collectively indicate that dillapiole affects implantation, as well as the *in vitro* potential of blastocysts to develop into post-implantation embryos.

### 2.4. Effects of Dillapiole on Developmental Potential of Blastocysts in Vivo

**Figure 4:**
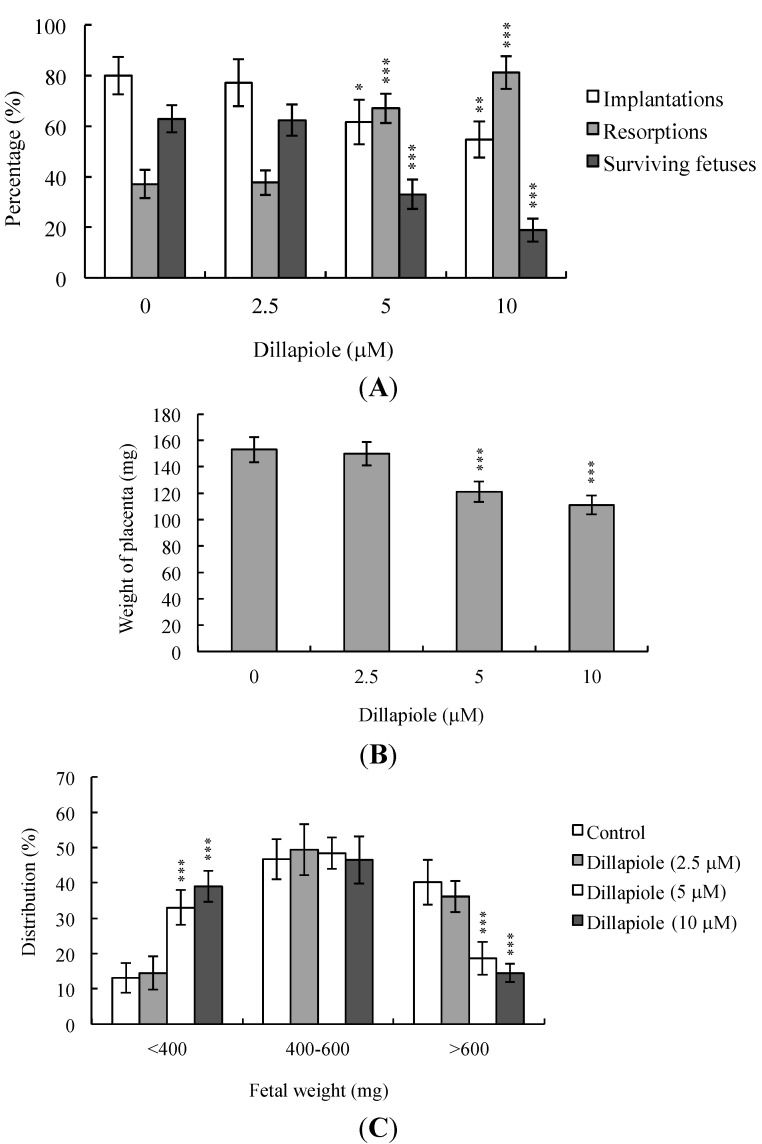
Effects of dillapiole on *in vivo* implantation, resorption, fetal survival and weight of mouse blastocysts. (**A**) Mouse blastocysts were treated with dillapiole (2.5, 5, or 10 μM) or left untreated for 24 h. Implantations, resorptions, and surviving fetuses were analyzed as described in Materials and Methods. Results are expressed as percentages representing the number of implantations, resorptions or surviving fetuses per number of transferred embryos ×100. Data are based on 360 total blastocysts across 45 recipients; (**B**) Placental weights of 45 recipient mice were measured; (**C**) Weight distribution of surviving fetuses at day 18 post-coitus. Surviving fetuses were obtained via embryo transfer of control and dillapiole-pretreated blastocysts, as described in Materials and Methods (360 total blastocysts across 45 recipients). * *p* < 0.05, ** *p* < 0.01, and *** *p* < 0.001 *versus* the control group.

To establish whether dillapiole affects blastocyst development *in vivo*, untreated control and dillapiole-pretreated mouse blastocysts were transferred, and the uterine content examined at 13 days post-transfer (day 18 post-coitus). The implantation ratio of the 2.5 μM dillapiole-pretreated group was not significantly different from that of untreated control mice, but was markedly decreased in the 5–10 μM dillapiole-treated group (Figure 4A). Embryos that implanted but failed to develop were subsequently resorbed. The proportion of implanted embryos that failed to develop normally was markedly higher in the group treated with 5–10 μM dillapiole (Figure 4A). Moreover, placental and fetal weights were lower in the dillapiole-treated group (Figure 4B,C). Previous studies, including a recent investigation by our laboratory, showed that 35%–40% of fetuses weigh more than 600 mg, and the average weight of total surviving fetuses is ~600 ± 12 mg in the untreated control group at day 18 of pregnancy in a mouse embryo transfer assay [13,19,28,29,30]. Fetal weight is an important indicator of developmental status. Accordingly, we employed the average fetal weight of the untreated control group as a key indicator of development of blastocysts treated with dillapiole. Notably, only 14.5% of the fetuses in the 10 μM dillapiole-pretreated group weighed more than 600 mg (an important marker of successful embryonic and fetal development), compared with 40.2% in the control group (Figure 4C). Clearly, dillapiole (10 μM) exposure at the blastocyst stage reduces embryo implantation and the potential for post-implantation development.

**Figure 5:**
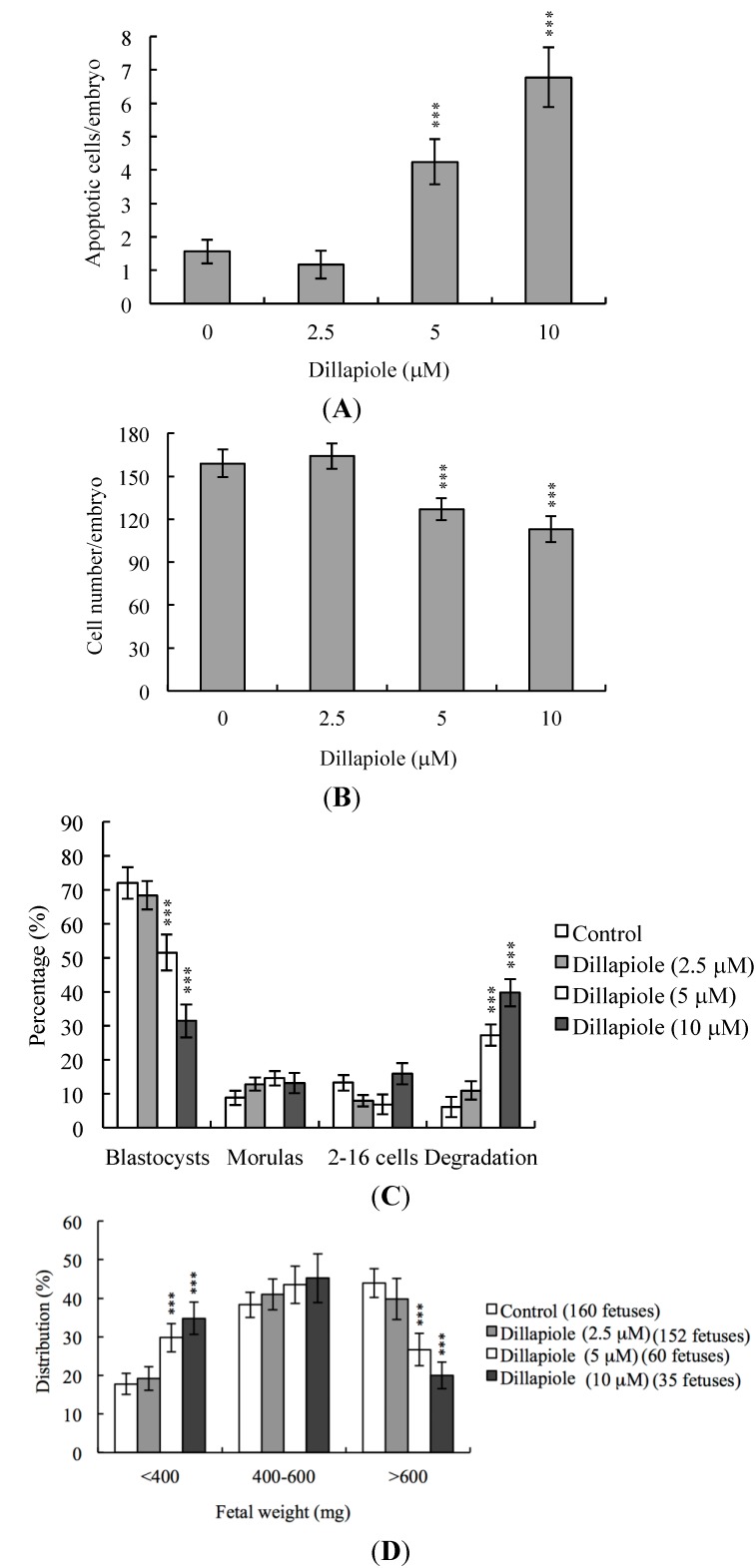
Effects of dietary dillapiole on apoptosis and blastocyst development in an animal model. For the duration of the experiment, randomly selected female mice were fed a standard diet and drinking water supplemented with or without dillapiole (2.5, 5, or 10 μM). After 24 h, female mice were mated overnight with a single fertile male of the same strain, and drinking water continuously supplemented with dillapiole (2.5, 5, or 10 μM) for four days. Blastocysts were obtained by flushing the uterine horn on day 4 after mating. (**A**) Apoptosis of mouse blastocysts was examined via TUNEL staining followed by light microscopy, and the mean number of apoptotic (TUNEL-positive) cells per blastocyst calculated; (**B**) Total numbers of cells per blastocyst were counted; (**C**) Developmental stages were compared in embryos obtained from mouse uterine horns on day four. Data are presented as the percentage of total embryos obtained; (**D**) Blastocysts were transferred to the uterine horns of day-four pseudopregnant mice. The surrogate mice were killed on day 18 post-coitus. The weights of the surviving fetuses and placenta were measured immediately after dissection. *** *p* < 0.001 *versus* the untreated control group.

Next, we examined the possible adverse effects of exposure to dillapiole on blastocyst development in an animal model. Female mice were fed a standard diet and drinking water either containing or devoid of dillapiole. Dillapiole consumption induced apoptosis to a significant extent and suppressed cell proliferation in mouse blastocysts (Figure 5A,B). In addition, dillapiole inhibited embryonic development to the blastocyst stage, causing embryo degradation (Figure 5C). Fetal weight was lower in dillapiole-treated than control animals (Figure 5D).

## 3. Discussion

During the complex and precisely orchestrated process of embryonic development, chemical or physical injury can affect normal progression and lead to malformation or miscarriage of the embryo. Thus, it is crucial to ascertain the possible teratogenic effects of various agents, including the phenylpropanoid, dillapiole, the main component of *Piper aduncum* that is also present in other Piper species. A recent study demonstrated for the first time that dillapiole induces cell death through the mitochondria-dependent apoptotic pathway by triggering oxidative stress, and exerts anti-proliferative effects on MDA-MB-231 cells. The broad cytotoxic effects of dillapiole against a variety of tumor cells were highlighted [11]. Safrole, a phenylpropanoid structure related to dillapiole also identified in *Piper* (genus) [20], is reported to trigger apoptosis in human oral cancer cells [25], as well as cell cycle arrest and apoptosis in human leukemia cells via endoplasmic reticulum (ER) stress-involved cell regulatory signaling cascade [26]. These findings support the potential of dillapiole for development as a chemopreventive and/or chemotherapeutic agent in human cancers. Experiments to determine the cytotoxic effects of 2.5–10 μM dillapiole on pre- and post-implantation embryonic development disclosed that dillapiole induces apoptosis, retards early-stage embryonic development, and negatively affects post-implantation progress (Figure 1, Figure 2, Figure 3, Figure 4 and Figure 5). TUNEL staining revealed that dillapiole triggers apoptosis in mouse blastocysts in a dose-dependent manner (Figure 1). Data from dual differential staining further showed that dillapiole-induced cell loss occurs primarily in the ICM (Figure 2). Our collective findings clearly indicate that dillapiole at a concentration range of 5–10 μM induces apoptosis and negatively affects mouse embryonic development, both *in vitro* and *in vivo* (Figure 1, Figure 2, Figure 3, Figure 4 and Figure 5).

TE arises from the trophoblast at the blastocyst stage and develops into a sphere of epithelial cells surrounding the ICM and blastocoel. These cells contribute to the placenta, and are required for development of the mammalian conceptus [31], meaning that a reduction in TE cell lineage may lead to decreased implantation and embryonic viability [32,33]. Interestingly, in our experiments, dillapiole induced cell loss in the ICM, but not TE, and had deleterious effects on the rate of implantation *in vivo* (Figure 2 and Figure 4). Previous studies have reported that ≥30% reduction in the number of ICM cells is associated with high risk of fetal loss or developmental injury, even in cases where implantation rate and TE cell numbers are normal [34]. In addition, the ICM cell number is essential for proper implantation, and reduction in the ICM lineage may decrease embryonic viability [32,33]. Apoptosis is responsible for eliminating unwanted cells during normal embryonic development but does not normally occur at the blastocyst stage [15,16]. Excessive apoptosis before or during the blastocyst stage may result in the deletion of important cell lineages, impacting embryonic development, and potentially leading to miscarriage or embryonic malformation [17]. In view of our observation that dillapiole (5–10 μM) reduces the cell number in the ICM of mouse blastocysts but has no effect on TE, we further investigated the possibility that the compound causes mortality and/or developmental delay in postimplantation mouse embryos *in vitro* and *in vivo* (Figure 2, Figure 4 and Figure 5). Dillapiole treatment of blastocysts led to decreased embryonic development and increased embryonic death *in vitro* and *in vivo* (Figure 3, Figure 4 and Figure 5).

Previous reports have demonstrated cytotoxic effects of dillapiole, with induction of cell apoptosis in MDA-MB-231 cells [11]. In addition, dillapiole has gastroprotective activity against ethanol-induced gastric lesions in Wistar rats. Recently, dillapiole was identified as the most active gastroprotective agent of *Peperomia pellucida* [35], supporting its utility as an effective anticancer or gastroprotective drug. Under these circumstances, safety is an important issue, especially in cases where the drug is used to treat pregnant women. Our results clearly indicate that dillapiole negatively affects mouse embryonic development. However, further research is required to ascertain the influence of dillapiole on human embryonic development. Moreover, we also suggest two possibilities regarding how mouse embryos expose to dillapoile *in vivo*. First, dillapiole or its metabolites in blood exert cytotoxic effects on oocytes to cause sequent pre-implantation embryonic injury. Second, dillapiole or its metabolites are secreted or diffuse to the oviduct or uterus to cause hazardous effects on embryonic development from the zygote to blastocyst stage. Either one or both mechanisms may cause impairment of mouse embryonic development *in vivo*. Experiments to confirm these hypotheses will be designed and performed by coworkers in our laboratory.

## 4. Experimental Section

### 4.1. Materials

Pregnant mare’s serum gonadotropin (PMSG), Bovine serum albumin (BSA), sodium pyruvate and dillapiole were purchased from Sigma (St. Louis, MO, USA). Human chorionic gonadotropin (hCG) was obtained from Serono (NV Organon Oss, The Netherlands). The TUNEL *in situ* cell death detection kit was obtained from Roche (Mannheim, Germany) and CMRL-1066 medium was from Gibco Life Technologies (Grand Island, NY, USA).

### 4.2. Collection of Mouse Morulas and Blastocysts

ICR mice were from National Laboratory Animal Center (Taipei, Taiwan). The animal study was approved by Institutional Animal Care and Use Committee (IACUC) of Chung Yuan Christian University (Permit Number: 10020) (Chung Li, Taiwan). All animals received humane care, as outlined in the Guidelines for Care and Use of Experimental Animals (Canadian Council on Animal Care, Ottawa, ON, Canada, 1984). All mice were maintained on breeder chow (Harlan Teklad chow) with food and water available *ad libitum*. Housing was in standard 28 cm × 16 cm × 11 cm (height) polypropylene cages with wire-grid tops and kept under a 12 h day/12 h night regimen. Nulliparous females (6–8 weeks old) were superovulated by injection of 5 IU PMSG followed 48 h later by injection of 5 IU hCG, and then mated overnight with a single fertile male of the same strain. The day a vaginal plug was found was defined as day 0 of gestation. Plug-positive females were separated for experimentation. Morulas were obtained by flushing the uterine tubes on the afternoon of gestation day 3, and blastocysts were obtained by flushing the uterine horn on day 4; in both cases the flushing solution consisted of CMRL-1066 culture medium containing 1 mM glutamine and 1 mM sodium pyruvate. Expanded blastocysts from different females were pooled and randomly selected for experiments.

### 4.3. Analysis of Developed Blastocysts from Morulas

Morulas were obtained by flushing the uterine tubes on the afternoon of gestation day 3 and treated with dillapiole (2.5, 5, or 10 μM) or left untreated for 24 h and cultured for an additional 24 h at 37 °C. Blastocysts were counted and percentages calculated using phase-contrast microscopy (Olympus BX51, Tokyo, Japan).

### 4.4. Dillapiole Treatment and TUNEL Assay

Blastocysts were incubated in medium containing the indicated concentrations of dillapiole (dissolved in dimethyl sulfoxide) for 24 h. For apoptosis detection, embryos were washed in dillapiole-free medium, fixed, permeabilized and subjected to TUNEL labeling using an *in situ* cell death detection kit (Roche Molecular Biochemicals, Mannheim, Germany) according to the manufacturer’s protocol. Briefly, each group of embryos was incubated with 20 μL of a TUNEL reaction mixture (2 μL of enzyme solution and 18 μL of labeling solution containing fluorescein-conjugated nucleotides) for 30 min at 37 °C. Next, embryos were extensively washed with phosphate-buffered saline (PBS) containing 0.3% (*w*/*v*) BSA. Converted-Peroxidase solution (20 μL) was added to each group of embryos, followed by incubation for 30 min at 37 °C. Next, the embryos were extensively washed once more with PBS. Finally, 20 μL of DAB substrate solution was added to each group of embryos and incubation for 2 min at room temperature followed. Photographic images were obtained via fluorescence microscopy conducted under bright light.

### 4.5. Dillapiole Treatment and Cell Proliferation

Blastocysts were incubated with or without culture medium containing 2.5, 5, or 10 μM dillapiole. After 24 h they were washed with s dillapiole-free medium and dual differential staining was used to facilitate counting of cell numbers in the inner cell mass (ICM) and trophectoderm (TE) [32]. Blastocysts were incubated in 0.4% pronase in M_2_-BSA medium (M_2_ medium containing 0.1% bovine serum albumin) for removal of the zona pellucida. The denuded blastocysts were exposed to 1 mM trinitrobenzenesulphonic acid (TNBS) in BSA-free M_2_ medium containing 0.1% polyvinylpyrrolidone (PVP) at 4 °C for 30 min, and then washed with M_2_ medium [36]. The blastocysts were further treated with 30 μg/mL anti-dinitrophenol-BSA complex antibody in M_2_-BSA at 37 °C for 30 min, and then with M_2_ medium supplemented with 10% whole guinea-pig serum as a source of complement, along with 20 μg/mL bisbenzimide and 10 μg/mL propidium iodide (PI), at 37 °C for 30 min. The immunolysed blastocysts were gently transferred to slides and protected from light before observation. Under UV light excitation, the ICM cells (which take up bisbenzimidine but exclude PI) appeared blue, whereas the TE cells (which take up both fluorochromes) appeared orange-red. Since multinucleated cells are not common in preimplantation embryos [37], the number of nuclei was considered to represent an accurate measure of the cell number.

### 4.6. Morphological Analysis of Embryonic Development

Blastocysts were cultured according to a modification of the previously reported method [38]. Briefly, embryos were cultured in 4-well multidishes at 37 °C. For group culture, four embryos were cultured per well. The basic medium consisted of CMRL-1066 supplemented with 1 mM glutamine and 1 mM sodium pyruvate plus 50 IU/mL penicillin and 50 mg/mL streptomycin (hereafter called culture medium). For treatments, the embryos were cultured with the indicated concentrations of dillapiole for 24 h in serum-free medium. Thereafter, the embryos were cultured for 3 days in culture medium supplemented with 20% fetal calf serum, and for 4 days in culture medium supplemented with 20% heated-inactivated human placental cord serum, for a total culture time of 8 days from the onset of treatment. Embryos were inspected daily under a phase-contrast dissecting microscope, and developmental stages were classified according to established methods [39,40]. Under these culture conditions, each hatched blastocyst attached to the fibronectin and grew to form a cluster of ICM cells over the trophoblastic layer via in a process called TE outgrowth. After a total incubation period of 96 h, morphological scores for outgrowth were estimated. Growing embryos were classified as either “attached” or “outgrowth”, with the latter defined by the presence of a cluster of ICM cells over the trophoblastic layer. As described previously [41,42], ICM clusters were scored according to shape, ranging from compact and rounded ICM+++ to a few scattered cells (+) over the trophoblastic layer.

### 4.7. Blastocyst Development Following Embryo Transfer

To examine the ability of expanded blastocysts to implant and develop *in vivo*, the generated embryos were transferred to 45 recipient mice. ICR females (white skin color) were mated with vasectomized males (C57BL/6J; black skin color; from National Laboratory Animal Center, Taipei, Taiwan) to produce pseudopregnant dams as recipients for embryo transfer. To ensure that all fetuses in the pseudopregnant mice came from embryo transfer (white color) and not from fertilization by C57BL/6J (black color), we examined the skin color of the fetuses at day 18 post-coitus. To assess the impact of dillapiole on postimplantation growth *in vivo*, blastocysts were exposed to 0, 2.5, 5, and 10 μM dillapiole for 24 h, and then 8 embryos were transferred in parallel to the paired uterine horns of day 4 pseudopregnant mice. The surrogate mice were killed on day 18 post-coitus, and the frequency of implantation was calculated as the number of implantation sites per number of embryos transferred. The incidence rates of resorbed and surviving fetuses were calculated as the number of resorptions or surviving fetuses, respectively, per number of implantations. The weights of the surviving fetuses and placenta were measured immediately after dissection.

### 4.8. Collection of Blastocysts from Female Mice Fed Drinking Water Containing Dillapiole

For the duration of the experiment, randomly selected 20 female mice were fed a standard diet and drinking water either supplemented with dillapiole (2.5, 5, or 10 μM) or left untreated. After 24 h, female mice were mated overnight with a single fertile male of the same strain, and allowed drinking water supplemented with dillapiole (2.5, 5, or 10 μM) for 4 days. Blastocysts were obtained by flushing the uterine horn on day 4 after mating. Blastocysts were transferred to the uterine horns of day 4 pseudopregnant mice. The surrogate mice were killed on day 18 post-coitus. The weights of the surviving fetuses and placenta were measured immediately after dissection.

### 4.9. Statistics

The data were analyzed using one-way ANOVA and *t*-tests and are presented as the mean ± SEM, with significance at *p* < 0.05.

## 5. Conclusions

In summary, we have reported for the first time that dillapiole induces apoptosis in the ICM of mouse blastocysts, leading to decreased embryonic development and viability. Our findings indicate that the dillapiole is an injury risk factor for normal embryonic development. Further studies are required to elucidate the mechanism(s) by which dillapiole affects embryonic development and establish its possible teratogenic actions on human embryogenesis.
